# 819. Experience with Isavuconazole Use for Treatment of Invasive Fungal Infections

**DOI:** 10.1093/ofid/ofad500.864

**Published:** 2023-11-27

**Authors:** Vanessa Gow-Lee, Supavit Chesdachai, Courtney E Harris, Omar M Abu Saleh

**Affiliations:** Mayo Clinic, Rochester, Minnesota; Mayo Clinic, Rochester, Minnesota; Brigham & Women's Hospital, Boston, Massachusetts; Mayo Clinic Rochester, Rochester, Minnesota

## Abstract

**Background:**

The treatment of invasive fungal infections (IFI) is often complicated by drug-drug interactions, toxicities, and host comorbidities. Isavuconazole (ISA) has a favorable side effect profile and has FDA approval for invasive aspergillosis and mucormycosis, but there are fewer data on its efficacy for other microbes and non-pulmonary infections. We report our experience of ISA in IFI treatment, with a particular focus on non-pulmonary sites and other microbial etiologies.

**Methods:**

We identified patients who received ISA as part of IFI treatment from 1/1/2015–4/1/2020 at Mayo Clinic. We categorized them by organ involvement and by pathogens, and identified patterns of ISA use and clinical outcomes.

**Results:**

A total of 131 patients were identified. Similar to the existing literature, the majority were pulmonary infections (60%), with 12% invasive sinus infection, 12% central nervous system (CNS) infection, and 7% fungemia. Aspergillus was the most common pathogen (39%), yeast comprised 19%, dimorphic fungi 15%, Mucormycosis 12%, phaeohyphomycosis 5%, and other molds 3%.

ISA was used as secondary/salvage therapy in 89% of cases. The response rate varied by organ involvement and pathogen. ISA had complete/partial resolution or achieved stability in 57% of fungemia cases, 63% of invasive sinus infections, 64% of pulmonary infections, and 73% of CNS infections. Aspergillus species had a 67% response rate, with 72% response in yeast infections, 65% rate in dimorphic fungi, 53% for mucormycosis, and 86% in dematiaceous molds.

**Figure:**
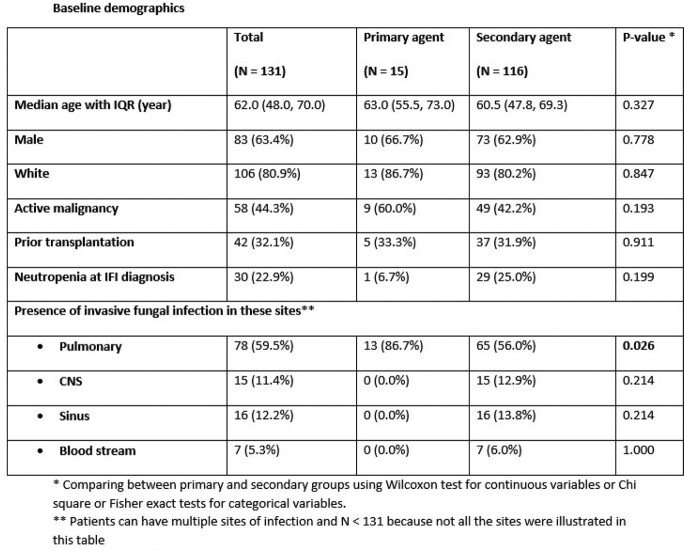

**Figure:**
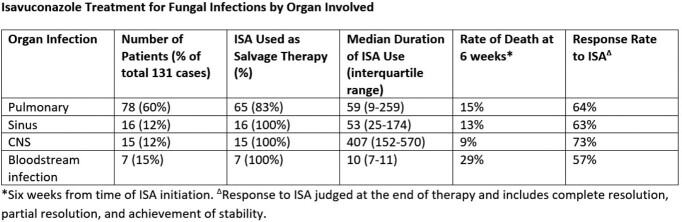

**Figure:**
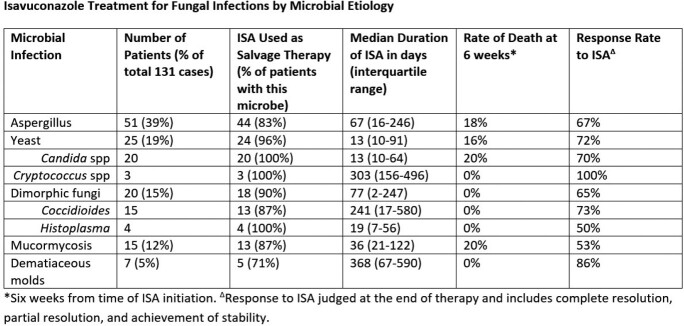

**Conclusion:**

Although limited by a small sample size, the subgroup review based on organ site and microbial etiology did seem to show roughly similar results to approved uses. The CNS infections treated with ISA were particularly of interest, given the limited options available with reliable CNS penetration.

The current available literature has little to say on the efficacy of ISA in these off-label uses, with the majority being similar case series. This report highlights the importance of prospective trials comparing ISA to other antifungals in a greater variety of indications to better identify when this better-tolerated agent can be safely used.

**Disclosures:**

**Courtney E. Harris, MD**, Dynamed: Advisor/Consultant

